# The Effects of Mindfulness on Brain Network Dynamics Following an Acute Stressor in a Population of Drinking Adults

**DOI:** 10.3390/brainsci16030312

**Published:** 2026-03-14

**Authors:** Shannon M. O’Donnell, W. Jack Rejeski, Mohammadreza Khodaei, Robert G. Lyday, Jonathan H. Burdette, Paul J. Laurienti, Heather M. Shappell

**Affiliations:** 1 Neuroscience Graduate Program, Wake Forest University Graduate School of Arts and Sciences, Winston-Salem, NC 27109, USA; shannon.odonnell@wfusm.edu; 2 Laboratory for Complex Brain Networks, Wake Forest University School of Medicine, Winston-Salem, NC 27109, USA; mohammadreza.khodaei@advocatehealth.org (M.K.); robert.lyday@advocatehealth.org (R.G.L.); jonathan.burdette@wfusm.edu (J.H.B.); heather.shappell@wfusm.edu (H.M.S.); 3 Department of Health and Exercise Sciences, Wake Forest University, Winston-Salem, NC 27109, USA; rejeski@wfu.edu; 4 Virginia Tech-Wake Forest School of Biomedical Engineering and Sciences, Wake Forest University School of Medicine, Winston-Salem, NC 27109, USA; 5 Department of Radiology, Wake Forest University School of Medicine, Winston-Salem, NC 27109, USA; 6 Department of Biostatistics and Data Science, Wake Forest University School of Medicine, Winston-Salem, NC 27109, USA

**Keywords:** fMRI, dynamic brain networks, hidden semi-Markov model, mindfulness, stress, drinking

## Abstract

**Highlights:**

**What are the main findings?**

- Participants that completed a guided mindfulness session following an acute stressor spent more time in a brain state in which the salience network was more active.
- Following an acute stressor, participants in the control group spent more time in brain states in which the default mode network was more active.

**What are the implications of the main findings?**

- Mindfulness may work to shift the brain out of states responsible for rumination and into a state that better supports emotional regulation and recovery following stress.
- This work offers a novel approach to testing and optimizing mindfulness-based therapies.

**Abstract:**

Background: Previous research has found that mindfulness-based techniques are beneficial for reducing stress in heavy-drinking individuals. However, the underlying neurobiology of these stress-reducing effects are unclear. Moreover, much of the research examining neurobiological correlates of mindfulness has used static functional connectivity, suggesting that brain activity goes unchanged for the entire length of an MRI scan. Methods: In the current study, we used a state-based dynamic functional connectivity model to examine brain states during either a 10 min mindfulness session or resting control that followed an individually tailored stress imagery task. Using a hidden semi-Markov model (HSMM), six brain states and the associated dynamics of state traversal were estimated for a population of moderate-to-heavy drinkers (N = 32). We modeled the 36 Schaefer atlas regions spanning the salience and default mode networks, and the HSMM characterized each state by its distinct multivariate pattern of activity and covariance structure. Group differences in dwell times, transition behavior, and overall state dynamics were evaluated using permutation tests and mixed-effects models. Results: Participants that experienced the mindfulness session had more transitions and longer time spent in states in which the salience network was more active. Participants assigned to the control group had more transitions and increased time spent in states in which nodes of the default mode network were more active. Moreover, for control participants, increased occupancy time to SN-dominant states was associated with lower perceived stress. Conclusions: Using HSMM provided a unique insight into network connectivity during mindful states; we believe it offers a novel approach to testing and optimizing mindful-based therapies.

## 1. Introduction

According to the 2024 National Survey of Drug Use and Health (NSDUH), approximately 222.8 million adults aged 18 and older reported that they drank alcohol at some point in their lifetime [1]. The self-medication hypothesis posits that individuals drink to cope with negative feelings and/or emotions and is a central theory in the etiology of alcohol use disorder (AUD) [2]. It is well known that stress is a key factor in alcohol consumption [3,4], as it increases the probability of alcohol use and amount of alcohol consumed [5,6,7]. Moreover, acute stress has been shown to contribute to the progression of drinking behaviors, from heavy drinking to alcohol abuse to alcohol dependence [8].

Two subnetworks of interest that are implicated in the pathophysiology of heavy drinking are the default mode network (DMN) and salience network (SN) [9]. The DMN includes regions such as the ventral medial prefrontal cortex (vmPFC), posterior cingulate cortex (PCC), and parts of the medial and lateral temporal cortex [10]. Evidence has suggested that the DMN is predominantly active at rest and during self-referential thought and introspection [11]. Altered activity and/or connectivity between the DMN and other cortical regions implicated in emotional regulation have been associated with increased stress-induced drinking [12]. Further, hyperactivation of the DMN has been linked to rumination, a response mode in which individuals repeatedly and passively focus on distress symptoms and the probable reasons for and outcomes of said symptoms [13]. Rumination is strongly correlated with depression and has been implicated as a vulnerability factor for substance use disorders [14]. The SN has two functional hubs, the insula and the dorsal anterior cingulate cortex (dACC), which are important in allocating attentional resources to the most salient stimuli [15]. The insula is a central component of the larger systems that process drug cues, stress, and reward [16,17]. Additionally, increased insular activity as a result of stress is associated with higher alcohol craving and consumption [18,19].

Emerging research has suggested that building resilience to stress could function as a protective factor against risky drinking behaviors [20,21]. In particular, following an encounter with an acute stressor, a viable action plan could be to invoke brief mindfulness-based stress reduction (MBSR) techniques [22]. The concept of mindfulness is defined as purposefully and non-judgmentally paying attention in a particular way to the present moment [23]. It is suggested that mindfulness techniques increase resilience to stress by enhancing cognitive awareness, which in turn leads to enhanced monitoring and processing of emotional responses [24]. Mindfulness practices have been used in a variety of settings, most notably to promote recovery from stress-induced alcohol seeking and relapse prevention [25,26,27,28,29]. The neurobiology underlying mindfulness-based stress reduction has uncovered two important subnetworks, the SN and the DMN [30]. During mindfulness, decreased activity of the DMN is associated with decreased mind-wandering and rumination, both of which contribute to anxiety and depression [31]. The SN is thought to play a key role in supporting mindfulness by directing attentional resources towards the present moment and suppressing any mind-wandering from the DMN [32].

However, much of the literature supporting the role of the DMN and SN in mindfulness is based on a paradigm of “static” connectivity. Static functional connectivity is calculated using correlations between blood oxygenation level-dependent (BOLD) signals [33] over the length of the entire MRI scan [34]. This type of analysis assumes that relationships between brain regions remain constant throughout a scan, which can range anywhere from 5–30 min. This may not be the case, especially during mindfulness, as participants can shift between various states of focus as well as distraction throughout a single session. Therefore, using models that assess dynamic functional connectivity provides a unique opportunity to investigate the changes in brain connectivity throughout a mindfulness session, avoiding masking of important results due to averaging data across a session. More specifically, using a model that estimates brain “states,” represented by unique brain networks of distinct activity and functional connectivity, has proved successful in identifying patterns of brain function associated with alcohol-related behaviors [35].

Following an acute laboratory stressor, the present study aimed to investigate dynamics of the DMN and SN during a mindfulness session versus a resting control in a randomized design to identify differences in state-based characteristics and explore possible correlates with self-reported stress. All participants were scanned following normal drinking behavior and following a 3-day abstinence period. As there was no difference between stress scores following the recovery scans between the normal and abstained condition, we aggregated the two sessions and input them into the model. The remainder of this paper focuses on the effects of mindfulness to reduce stress reactivity among individuals that are moderate-to-heavy drinkers, as defined in Section 2.1. Two hypotheses will be explored in this paper. First, we hypothesized that participants randomized to a brief guided mindfulness session following an acute stressor would have higher occupancy time in and transition frequencies into states in which nodes of the SN are more active when compared to the control group. Second, we hypothesized that those in the control group would have higher occupancy time in and transition frequencies into states in which nodes of the DMN are more active. Finally, we hypothesized that longer time spent and increased transitions to SN-dominant states would be associated with lower perceived stress following recovery.

## 2. Materials and Methods

### 2.1. Participants

Thirty-four individuals who were moderate-to-heavy drinkers were recruited from the local community using a variety of advertisement techniques, such as flyers, mailers, and internet postings. The final sample consisted of 32 participants, as two participants were removed. One was removed due to having stress scores far outside the range (more than double the mean of everyone else), causing them to be highly influential data points (i.e., they had a large influence on the association between stress score and brain state occupancy time). The other was removed as they had a high number of transitions early on in each of their scans, most likely due to motion, which caused them to have a large influence on group differences for occupancy time, transition frequency, and dwell time. For completeness, results that include this participant are included in the Supplementary Material. A priori analyses determined no significant differences in the stress scores based on assigned drinking state following the recovery period (*p* = 0.252). As such, both the normal- and abstained-drinking-state scans were included in subsequent analyses to increase statistical power.

To be included in this study, all participants had to drink at least 4 days/week. Female participants had to consume 1–3 drinks per day, and male participants had to consume 2–4 drinks per day, and all participants had to demonstrate these drinking behaviors for at least the past three years. According to a recent definition from the Centers for Disease Control, moderate alcohol use is defined as males consuming two drinks or less in a day and women consuming one drink or less in a day [36]. Thus, at a minimum, all participants met the moderate drinking criteria. The NIAAA defines heavy alcohol use as males consuming five or more drinks in a day and women consuming four or more drinks in a day [37]. Some participants also met this heavy drinking criterion. Therefore, throughout this document, we refer to the participants as having moderate-to-heavy drinking behavior. During a preliminary screening, drinking behavior was confirmed using responses to the first three questions of the standardized Alcohol Use and Disorders Identification Test (AUDIT) [38] and a modified version of the Timeline Follow-Back (TLFB) questionnaire [39] to include time of day. We excluded individuals that binged more than once a month, drank before noon more than four times in a month, or had a current/past clinical AUD diagnosis. However, we did not do a clinical evaluation. There is a possibility that, given the high levels of drinking, some of the participants may meet criteria for an AUD diagnosis. Other exclusion criteria included the following: current or previous diagnosis of psychiatric disorders, current neurological disorders, smoking > 30 cigarettes per day, consuming ≥ 500 mg of caffeine per day, and positive urine drug screening (methamphetamine, cocaine, marijuana, amphetamines, opiates, and benzodiazepines). Because of the association between body mass index (BMI) and blood-alcohol concentration (BAC), BMI was restricted to a range of 18.5–35 [40]. Participants also had to be right-handed, not claustrophobic, and have no contraindications to MRI.

### 2.2. Stress Imagery and Mindfulness

To develop scripts for the acute stress imagery session, participants completed an interview session to gather individualized descriptions of stressful stimuli. After these descriptions were collected, participants rated them using a 10-point Likert scale in which 0 = not at all stressful and 10 = the most stress they have felt in their life. Stimuli rated ≥8 were used for the stress imagery script development. The information obtained to create the imagery scripts was then used to create an audio recording for both of the MRI visits. Imagery scripts have been standardized and used previously as an effective technique for modeling stress in the laboratory. The scripts have also been validated via detection of increased cortisol following stressful imagery sessions [41,42]. While we did not measure cortisol in this study, we did use these methods as stressful imagery for the participants. For an example of both neutral and stressful scripts used in the present study, please refer to the Supplementary Material page.

Following the screening visit, participants were randomized to one of two 10 min protocols, a period of active mindfulness or a control period, which occurred immediately following the stressful imagery session in the scanner. The active mindfulness session involved subjects listening to a guided experience in mindfulness-based meditation using a script created by a co-investigator. The script focused on breath counting, accepting any negative feelings that may arise, and body centering. The control session involved subjects being instructed to lie quietly and rest.

### 2.3. MRI Study Visits

Each participant completed three visits: a baseline screening visit and two MRI visits. Following the initial phone screening, the baseline visit was conducted to obtain informed consent, verify negative drug and alcohol tests, collect self-report questionnaire data, and have participants complete the modified TLFB. The MRI visits were performed in a normal drinking state, defined as typical alcohol consumption for the past 3 days, and in an abstained drinking state, in which a 3-day period of abstinence was imposed. Participants were randomly assigned to either complete the abstained-state scan first or to complete the normal-state first.

Participants were provided a Breathometer (Breathometer Inc., company closed in 2017, Burlingame, CA, USA) or a BACtrack Mobile Pro (KHN Solutions Inc., www.bactrack.com, San Francisco, CA, USA) alcohol breath testing device to assess breath alcohol at home. They were also given an iPhone with the associated app for the device and were alerted randomly throughout the day to complete breath tests to ensure abstinence. The results were downloaded from the iPhones when participants came to the study session. In addition, participants were contacted one day prior to their scan session to confirm they were maintaining abstinence. At the study session, participants also had to complete a breath and urine sample collection to verify the absence of drugs and alcohol before beginning the scan. To screen for alcohol withdrawal syndrome, the Clinical Institute Withdrawal Assessment for Alcohol Scale, Revised [43] was completed by each participant upon arrival for the study visit on the abstinence day. A score exceeding 7 would terminate the session and initiate an IRB-approved protocol to help the participant obtain treatment. No participant in this study scored above 7.

### 2.4. Scan Session Protocol

**Figure 1:**
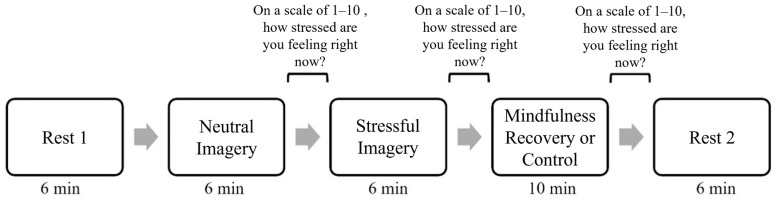
Visual representation of the functional imaging protocol used during the MRI scanning session. This full protocol was completed for each of the participants assigned drinking states.

The MRI scan protocol consisted of one high-resolution anatomical sequence followed by a series of functional scans with a 10-point Likert scale response in-between scans to assess stress levels, imagery vividness, and alcohol craving. After the anatomical sequence, a 5 min resting-state functional scan was performed. Next, a functional scan was performed while participants listened to the neutral image script. Immediately following this scan, participants were asked to rank their stress levels and the vividness of the imagery. Next, another functional scan was collected while participants were listening to their individualized stressful imagery scripts. Again, immediately following this scan, participants were asked to rank their stress levels, imagery vividness, and alcohol craving on a scale from 1–10. Next, participants completed either a 10 min guided mindfulness experience or were asked to lie quietly and rest for 10 min. Once more, participants were asked to rank their stress and alcohol craving on a scale of 1–10 immediately following the mindfulness/control sessions. These self-reported stress scores were used to examine associations between state-based characteristics during mindfulness and stress levels directly following the recovery period. Finally, another 5 min resting-state functional scan was performed. The full scanner protocol was completed twice for each participant, once in a normal drinking state and once in a period of imposed abstinence. For a visual representation of the functional portion of the scanning protocol followed in this study, see Figure 1.

### 2.5. MRI Data Acquisition and Processing

Functional and anatomical imaging data was acquired on a 3T Siemens Skyra Scanner (Siemens Healthineers, Malvern, PA, USA) with a 32-channel head coil, a rear projection screen, and MRI-compatible headphones. The imaging protocol involved a T1-weighted structural scan followed by a series of BOLD-weighted scans with varying lengths (Figure 1). For the current manuscript, we focused specifically on the mindfulness and control group recovery scans that followed the stress imagery. The high-resolution (1 mm isotropic) T1-weighted anatomical sequence was performed using a single-shot 3D MPRAGE GRAPPA2 sequence with a repetition time (TR) of 2.3 s, echo time (TE) of 2.99 ms and 192 slices. The BOLD-weighted image sequences were acquired using an echo-planar imaging sequence with a 3.5 mm × 3.5 mm × 5 mm resolution, a TR of 2 s, a TE of 25 ms, a flip angle of 75°, and 35 slices per volume. The recovery scans used here had 297 volumes.

Data was preprocessed using Statistical Parametric mapping (SPM12) [44]. Each participant’s T1 was coregistered to the Montreal Neurological Institute (MNI) 152 brain template [45] to enhance segmentation and warping. Unified segmentation of the T1 images provided warping parameters to the standard space MNI template as well as brain segment images of grey matter, white matter, and cerebrospinal fluid. For each functional scan, the first 10 image volumes were removed to allow the fMRI signal to reach a steady state. Then, slice timing correction and realignment were performed. Finally, the functional scans were coregistered with the subject’s T1 image and normalized to the MNI template using the previously mentioned warp. To remove low-frequency drift and high-frequency physiological noise, a band-pass filter of 0.009–0.08 Hz was applied. Six motion parameters and the average signal of grey matter, white matter, and cerebrospinal fluid were regressed out. Motion scrubbing was used to identify problematic volumes; bad volumes were labeled as 1 and then used as an additional regressor. This technique was used so as to not remove continuous time, which could lead to inaccurate estimates of the network state dynamics. Next, we extracted atlas-based preprocessed time series in standard space for each subject by calculating the mean of the preprocessed time series from each of the 36 Schaefer atlas regions [46] that make up the SN and DMN. These extracted time series were used as input for the MIND-Map toolbox [47] that uses a hidden semi-Markov model (HSMM) [48] to estimate brain states and dynamics. Lastly, three timepoints were removed from the beginning and end of the signal to correct for edge effects caused by band-pass filtering.

### 2.6. Hidden Semi-Markov Modeling (HSMM)

We applied a HSMM to the 36 Schaefer atlas regions that make up the SN and DMN from our final sample of study participants. The list of regions used is in the Supplementary Materials. While a detailed description of the HSMM is available in our previous work [38], we provide a brief summary here. For each participant, we denote the ROI time series for each participant by *Y_i_*_1_, …, *Y_iT_*, where each *36*-dimensional vector *Y_it_* contains the BOLD measurements of the 36 ROIs at the *tth* timepoint for the *ith* participant. We assume that each observed vector, *Y_it_*, is generated by an unobserved (hidden) brain network state. This hidden state at time *t* for participant *i* is denoted by *S_it_*, where *S_it_* takes on discrete values. That is, *S_it_* ∈ 1…*K*. Conditional on the current hidden state *S_it_*, the observed data *Y_it_* are modeled as following a multivariate normal distribution *Y_it_* ∼ N (*µ_s_*, Σ*_s_*), where the mean and covariance are dependent on the current (unknown) network states for any *S* ∈ 1, …, *K*.

Thus, each network state is characterized by a unique pattern of mean activity across ROIs, along with a distinct covariance (and corresponding correlation) structure. We convert each state’s covariance matrix to a correlation matrix, which serves as the weighted brain network representation for that brain state. Moreover, the HSMM includes parameters that model (a) the transition probabilities between network states and (b) the dwell time distributions for how long each state persists after it is entered. These dynamic properties were then compared between the mindfulness and control groups via permutation testing and mixed-effects models (refer to Section 2.5).

For our analyses, we estimated a single set of network states using data pooled from all participants. The number of states must be defined in advance and is strongly influenced by such factors as the number of participants, timepoints, and regions of interest (ROIs). This is because increasing the number of states also increases the number of parameters that need to be estimated. To identify the optimal number of states, we ran the model using a range of state numbers (3–7) and evaluated the Euclidean distances between the resulting states. For each run, we calculated the minimum distance between any pair of states, with higher values indicating greater distinctiveness. The six-state model exhibited the highest minimum distance, suggesting it provided the most clearly differentiated set of states (see the Supplementary Material for distance plots across runs). Moreover, for full details of the HSMM, including the complete data log-likelihood and parameter estimation routine, we direct readers to the Supplementary Material.

### 2.7. Statistical Inference and Permutation Testing

As described above, a priori analyses determined there were no significant differences between stress scores in either drinking state (normal versus abstained) immediately following the recovery period, so we focused our analyses on comparing state network dynamics between the mindfulness and control recovery groups. Thus, each participant’s normal and abstained state recovery scans were included in the model to increase the number of parameters and allow us to include more ROIs for the analyses.

We fit a single HSMM to the full sample to estimate the six latent states. Using the state, transition probability, dwell time density, and initial state probability estimates obtained from this model, we then estimated each participant’s most probable sequence of latent states using the Viterbi algorithm [49]. In other words, although the latent states themselves are estimated from the entire dataset and are not subject-specific, the Viterbi algorithm identifies when each of those states is active for a given participant based on their BOLD time series data. From each participant’s Viterbi-decoded state sequence, we derived three summary measures: (a) total occupancy time in each state, (b) transition frequency into each state, and (c) dwell times within each state. These represent different summaries derived from the same fitted HSMM.

Occupancy time for each state was calculated by summing the total number of timepoints spent in that state, dividing by the length of the participant’s time series, and converting it to a percentage. Group averages for each state were calculated by taking the mean across all participants within each group. Transition frequency was defined as the number of times a participant entered each state. Because each participant contributes one occupancy value and one transition count per state, these outcomes are well suited to mixed-effects modeling and allow for straightforward participant-level comparisons between these summary measures and participant stress scores. Therefore, we investigated whether occupancy times or transition frequencies were associated with stress scores. Specifically, we extracted the occupancy time for each state, as well as the transition frequency into each state, for each participant. We fit mixed-effects linear regression models separately for each state’s occupancy time and transition frequency, with stress score as the outcome. Along with the state-specific summary measure, each model included an indicator variable for group membership (mindfulness vs. control), an interaction term between group membership and the state measure, and the average number of drinks consumed in the prior 30 days. A random intercept was included to account for participants contributing two scans. Stratified models were conducted for any models with a significant interaction, such that separate models were fit within the mindfulness and control groups. All statistical analyses were corrected for multiple comparisons using the false discovery rate (FDR) [50].

Dwell time (the number of consecutive timepoints spent in a state) presents a different statistical challenge. Participants vary substantially in how many times they enter a given state: some individuals enter a state only once (yielding a single dwell time), whereas others enter the same state multiple times (yielding several dwell times). This results in an unequal and often small number of dwell time observations per participant, making it difficult to define a single, reliable participant-level summary suitable for mixed-effects modeling. We therefore only compared empirical dwell time distributions between groups rather than modeling dwell times at the participant level.

To calculate empirical dwell time distributions, we counted the number of consecutive timepoints spent in each state for each participant. These dwell times were then aggregated across all participants within each group, and a density estimate was computed for each state. As in the original analysis, we excluded the first and last dwell periods for each participant to avoid bias toward shorter dwell times, as the duration of time spent in a state immediately before or after the scan was unknown. After obtaining separate empirical dwell time distributions for each group across all six states, we quantified group differences using the Kullback–Leibler (KL) divergence, which measures the directed divergence between two probability distributions. A KL divergence of 0 indicates identical distributions, whereas values approaching 1 reflect substantial differences. Statistical significance for dwell time distribution differences was assessed using permutation testing with 500 permuted samples. For more details on KL divergence in this context, refer to [48].

Similarly, we also conducted permutation tests to investigate whether there were group differences in occupancy time and transition frequencies. For each participant, we computed state-specific occupancy time by summing the number of timepoints spent in a given state, dividing by the total length of the time series, and converting this value to a percentage. Group-level differences were obtained by subtracting the mean occupancy percentage of the control group from that of the mindfulness group, and this observed difference was evaluated against a null distribution generated from 500 label-permuted samples. Transition-frequency counts into each state were analyzed in an analogous manner: individual transition counts were summarized, group means were contrasted, and the observed group difference was compared with its corresponding permutation-based null distribution. Once again, all statistical analyses were corrected for multiple comparisons using the false discovery rate (FDR) [50].

### 2.8. Modularity Analysis for State Characterization

For each of the 6 brain states, we performed a modularity analysis to further understand each state’s defining topological characteristics. The modularity analysis identified community structures within each state. Modularity analysis estimates communities of nodes that have stronger within-group connections than their between-group connections [51]. As a result, information flow is higher within communities compared to between communities. We applied the Newman spectral community detection method to the positive network of each state [52]. A gamma value of 1 was used to divide the networks into more communities.

## 3. Results

### 3.1. Descriptive Statistics

Demographic and alcohol use characteristics for the sample can be found in Table 1. There were a total of 32 participants (19 female), 15 of which were assigned to the mindfulness protocol while 17 were assigned to the control group protocol. Of the full sample, 87.5% identified as White, 9.4% identified as African American or Black, and 3.1% identified as Asian, and the sample averaged 38.2 years of age. There were no significant differences between the mindfulness and control groups for the following demographic variables: age, race, sex, age of first drink, total years drinking, and percentage of drinking days in the past month. There was a significant difference in the average number of drinks on drinking days between the mindfulness and control groups (*p* = 0.02), with the control group averaging 2.5 drinks per day and the mindfulness group averaging 1.9 drinks per day. Therefore, we adjusted for this variable in our mixed-effects models. In these models, the average number of drinks did not significantly predict stress scores. Additionally, there was a significant mean difference (*p* = 0.03) in the 10-point stress scales taken immediately after the recovery period, with participants in the mindfulness group reporting lower perceived stress (M = 1.4, SD = 1.36) than those in the control group (M = 2.3, SD = 1.52). A bar plot depicting the mean ± SD of perceived stress scores can be found in Supplementary Figure S1.

### 3.2. Functional Brain State Characteristization

**Figure 2:**
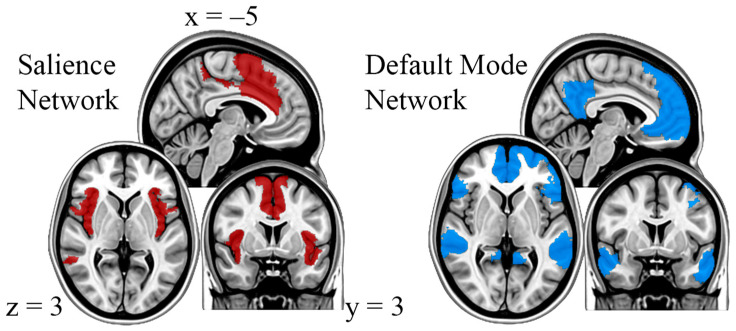
Localization of the salience network (SN, red) and default mode network (DMN, blue) as defined by the Schaefer 100-node atlas.

**Figure 3:**
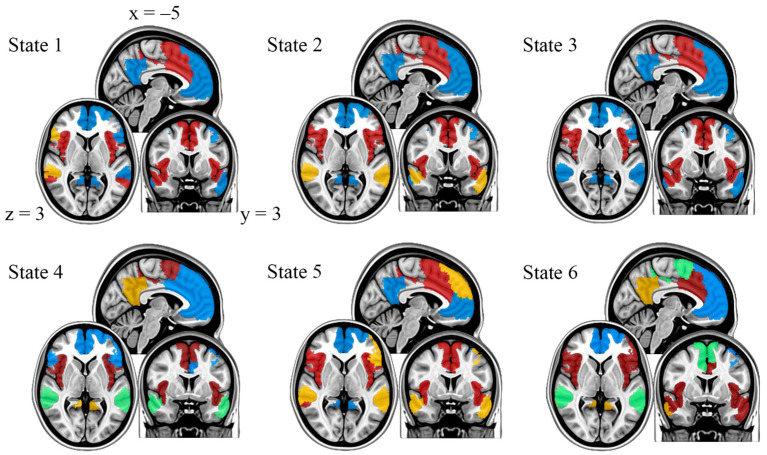
Module assignments for each state, represented by color grouping. Colors are matched as close as possible to the predefined SN and DMN. Differences in the color assignments in the modular maps across states reveal the “splitting” of nodes from either the DMN or SN.

A reference image of the DMN and SN as well as node community assignments, or modules, for each state can be found in Figure 2 and Figure 3, respectively. The only state to have nodes belonging to the DMN and SN parcellated into two distinct modules was state 3. States 1, 2, and 5 had three modules, while states 4 and 6 had four. For states 1 and 2, nodes of the SN remained in a module together, and the temporal regions of the DMN formed a module, shown in yellow. In state 5, the SN remained intact as well, but the DMN was split into two modules: one module consisting of bilateral temporal/parietal regions and the dorsolateral PFC in yellow and another module containing the PCC and ventromedial PFC (vmPFC) in blue. In state 4, the SN remained intact, but the DMN formed three distinct modules: the prefrontal cortex (PFC) in blue, left and right temporal regions in green, and the PCC in yellow. In state 6, both the DMN and SN were split into four communities: anterior/posterior DMN in blue and yellow and anterior/posterior SN in red and green.

**Figure 4:**
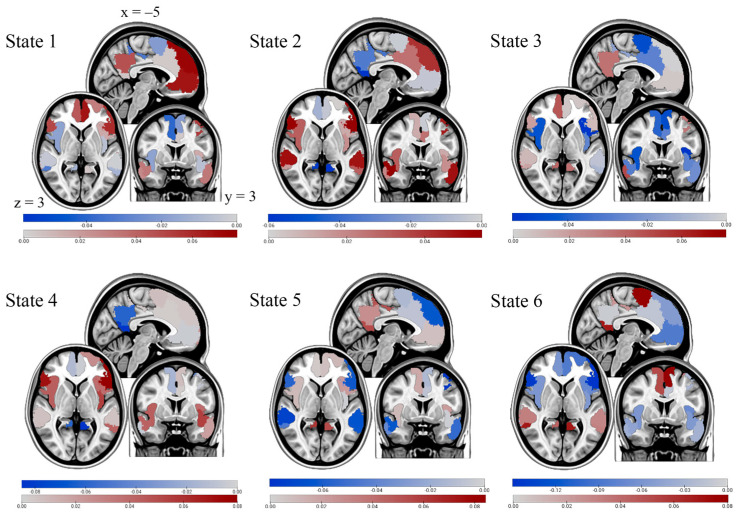
Mean activation maps for each state. Dark blue colors indicate lower activity, grey/white colors indicate average activity, and dark red colors indicate higher activity across states. Activity is relative to that observed during the other states, not relative to a baseline.

Mean activity maps are represented by the mean z-scored BOLD signal for nodes in each state compared to activity in the other states (Figure 4). In state 1, anterior DMN nodes are primarily active, including the left prefrontal cortex and temporal/parietal regions. States 3, 5, and 6 are characterized by high posterior DMN activity. Specifically, the posterior cingulate cortex, a hub of the DMN, displays high activity across all three states. In state 2, many SN nodes show high activity, specifically the insula and the dorsal anterior cingulate cortex (dACC), both of which are hubs within the SN. Finally, state 4 is characterized mainly by high activity in the bilateral insula.

### 3.3. Occupancy Time, Transition Frequency, and Dwell Time

**Figure 5:**
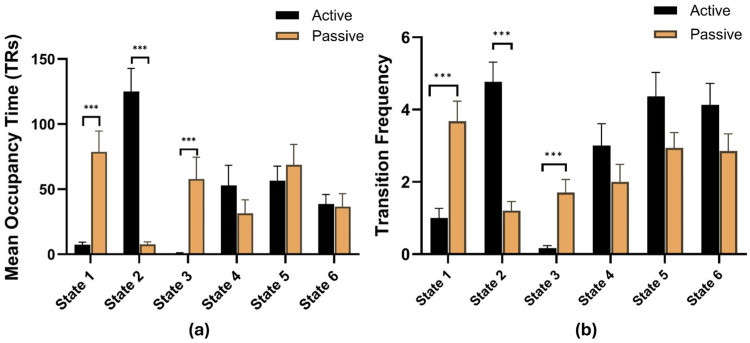
(**a**) Mean occupancy time, represented by average number of TRs, for each cohort in each state. (**b**) Transition frequency for each cohort to each state. States 1 and 3 were characterized by high DMN activity. State 2 was characterized by high SN activity. *** *p* < 0.001.

Participants in the mindfulness group spent significantly more total scan time, on average, in state 2 during the recovery period (*p* = 0.0002) compared to the control group. Furthermore, participants in the mindfulness group transitioned significantly more frequently into state 2 (*p* = 0.0002) throughout the recovery period compared to participants in the control group. Conversely, participants in the control group spent significantly more time in states 1 and 3 during the recovery period (*p* = 0.0002, *p* = 0.0002) and had significantly more transitions into states 1 and 3 (*p* = 0.0002, *p* = 0.0002) compared to the mindfulness group. The results of the permutation test for each state and each state characteristic can be found in Table 2. Bar plots depicting occupancy time and transition frequency for each cohort in each state can be seen in Figure 5.

**Figure 6:**
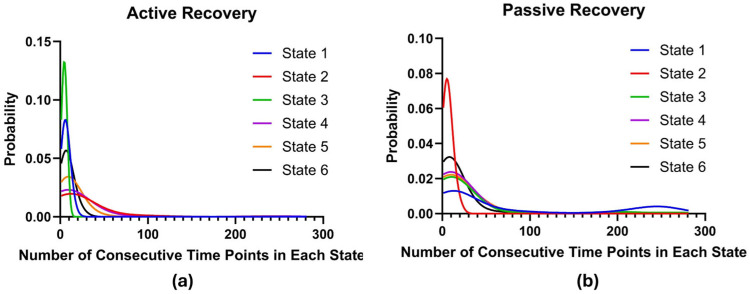
Estimated dwell time distributions for each state for the mindfulness (**a**) and control (**b**) recovery groups.

Dwell (sojourn) time represents how many consecutive time points a participant spends in a state before transitioning to another. There was a significant difference in sojourn distributions between the two groups for states 1, 2, and 3 (*p* < 0.0001, *p* < 0.0001, *p* = 0.004). Specifically, we found that individuals in the mindfulness group had shorter dwell times in states 1 and 3 and a longer dwell time in state 2 before switching to another state. Participants in the control group had a shorter dwell time in state 2 and a longer dwell time in states 1 and 3 before switching to another state. Sojourn distributions for the six states for each recovery group can be found in Figure 6. Higher curves to the left of the *x*-axis represent shorter dwell times for that state.

### 3.4. Mixed-Effects Model and Stress Scores

The results from the repeated-measures mixed-effects model determined there was an association between brain state dynamics and stress scores immediately following the recovery period. Interestingly, we found that increased occupancy time in state 2 was significantly associated with lower stress scores in the control group (*p* = 0.002) following the recovery period (Table 3). No other associations between state occupancy time and stress scores, as well as transition frequency to the other five states and stress scores, were found. Additionally, there were no significant effects found when examining the association between total transition frequency (across all five states) and stress scores.

## 4. Discussion

Following acute stress, we examined the effects of mindfulness on brain network dynamics of individuals who were moderate-to-heavy drinkers. We determined optimal state(s) for both the guided mindfulness and control groups and investigated how each group moved through these states. Additionally, we determined which dynamic characteristics were associated with either higher or lower stress scores for each group. Specifically, we found that six optimal states emerged from a dynamic analysis of brain states during the recovery period, supporting our hypotheses that, compared to the control group, guided mindfulness yielded higher occupancy time and transition frequency groups to states in which nodes of the SN were more active; conversely, the control group exhibited higher occupancy time and transition frequency to states in which nodes of the DMN were more active. We also observed lower perceived stress following the recovery period among participants in the mindfulness group as compared to the control group and a significant correlation between occupancy time and stress scores following the recovery period in the control group. Below, we discuss possible interpretations of our findings.

The mindfulness group spent longer periods of time, consecutively and on average, in state 2. Moreover, individuals in the mindfulness group transitioned into this state more often than the control group. Modularity analysis of state 2 revealed unique patterns of functional connectivity, with three distinct modules emerging. Nodes of the SN formed a module together, temporal nodes of the DMN formed another, and the PFC and PCC formed a third module. Activity maps revealed increased activity in the SN module, the temporal DMN module, and the dorsomedial PFC (dmPFC), part of the larger PFC-PCC module, whereas the remainder of the PFC-PCC module showed low-to-normal activity relative to the other five states. The fact that participants in the mindfulness group spent more time in state 2 was not surprising, as several studies have implicated increased activity of the SN as a key mechanism underlying positive therapeutic effects of mindfulness-based interventions [53,54,55]. The fact that participants in the control group spent more time in states characterized by increased activity in modules of the DMN, implicated in rumination, offers strong support for the beneficial role of mindfulness-based recovery.

Interestingly, as noted in the modularity analysis, there were also two distinct modules of the DMN that were active in state 2: the temporal nodes of the DMN and the dmPFC. Recent work has observed increased activity in cortical regions of the temporal lobe of the DMN, specifically within the context of processing information related to the self [56]. While this may seem counterintuitive to the positive effects of mindfulness, it is important to emphasize that paying attention to and processing interoceptive cues, which was a central feature of the mindfulness script, is known to enhance a more integrated perspective of the self via the right hemisphere [57], and this is the basis for mindful awareness body therapy that was designed specifically to address dysfunction in emotional regulation among participants with alcohol abuse disorders [58]. Additionally, the increase in dmPFC activity seen in state 2 is consistent with findings showing an increase in dmPFC activity during meditation, most likely because of its role in cognitive emotional evaluation and regulation [54,59].

Hence, we conclude that the mindfulness condition—specifically increased activity in nodes of the temporal DMN, dmPFC, and SN—were working in concert to maintain an integrated sense of self in present moment awareness [59]. It is known that allocating attention to interoceptive cues is dependent upon the SN [53,54,55], plays a major role in the right hemisphere’s construction of an integrated sense of self [57], is a central feature of mindful-based training programs [22], and likely plays a role in the experience of mindfulness-based transcendent states [60]. By disengaging areas of the brain responsible for alcohol craving and consumption, mindfulness may serve as a feasible strategy when faced with stress [61]. Furthermore, activating brain regions responsible for emotional regulation and present moment attention may help curb stress-induced alcohol consumption [62].

States 1 and 3 were significant for the control group in that subjects transitioned more often into these states and spent longer consecutive and overall timepoints in these two states compared to the mindfulness group. Modularity analysis for state 3 determined that nodes of the SN formed a module together, while nodes of the DMN assembled into another module. In this state, the PCC, a hub of the DMN, exhibited increased activation relative to the other states. State 1 connectivity patterns were unique in that most nodes of the PFC, PCC, and left temporal nodes of the DMN were in a module together, nodes of the SN were in another module, and the right temporal and ventral PFC (vPFC) were in a third module. Activation maps for state 1 were characterized by increased activity of the PCC and PFC, while state 3 activity was localized to the PCC only, relative to the remaining states. Increased activity of hubs within the DMN and decreased activity of the SN, as seen in states 1 and 3, is consistent with previous work establishing the role of this subnetwork as being active during periods of rest and may even be implicated in mind-wandering and rumination [63]. Further, this supports previous work that has observed these two subnetworks exhibiting anticorrelated activity at rest [64]. As described above, the control was established as a rest period to be used as a comparison to the mindfulness group. Thus, as hypothesized, the control group spent more time engaged in DMN activity known to be associated with rumination and less time in SN activity relative to the mindfulness group.

The current study has some limitations to note. First, the mindfulness session used in this study was a one-time, 10 min, guided experience with no training specific to the intervention prior to the study. Although single bouts of mindfulness have proved successful in reducing stress and stress-related alcohol consumption and craving [26,65], studies have shown that the more experienced one is in meditation, the more likely they are to build resilience to stress and be more successful in coping with stress in the future [29]. Hence, an important extension of this study would be to expose participants to formal training in mindfulness, to have them employ skills in response to acute stressors across the day, and to evaluate the effects of this training using both experience sampling methodology and controlled study in a laboratory setting. We did not collect data on whether the participants had previous experience with mindfulness training. Future studies should take this into account, as participants with more mindfulness training may be more likely to recover from stress quickly.

Our analysis estimated that six states were the best fit for the HSMM model, as these six states produced the most stable state sequences across the sample. However, participants may enter more (or fewer) than six states during a 10 min mindfulness session scan. Further, ROIs used were cortical nodes of the SN and DMN, thus possibly missing important contributions of subcortical structures and the central executive network (CEN), which has also been a network of interest during mindfulness [66]. Future methodological work in this area should allow for larger state sizes (i.e., more brain regions to be included in the analyses), as well as a way to quantify whether the number of and estimate of the network states fits each individual well.

Additionally, we employed a single item of perceived stress following the mindfulness and control group conditions. In hindsight, it would have been beneficial to have more comprehensive assessments of stress and affect following recovery. We highly recommend these additional assessments in future study designs. We also acknowledge the confounds between the mindfulness and control conditions, particularly the fact that there is a breathing exercise and an auditory stimulus in the mindfulness group but not in the control group. Changes in breathing patterns have been shown to impact BOLD signal fluctuations as a source of noise [55]; however, breathing exercises were included in this study as they are a central component of mindfulness practices. In the future, including physiological measures, such as galvanic skin response (GSR), heart rate variability (HRV), and cortisol levels in the mindfulness condition, would be beneficial in attempting to disentangle this confound.

We acknowledge a major limitation in this study. The mindfulness condition involved an auditory stimulus, which is potentially the result of the observed differences between the mindfulness and control groups. Further, the DMN is known to decrease activity when a stimulus requiring active engagement is present compared to a rest condition in which participants are not being presented with a stimulus [67]. Put simply, the DMN-related findings in this study may be a result of participants simply participating in a task being compared to participants in a rest condition rather than a direct effect of mindfulness. In the future, we recommend that mindfulness studies include a third, neutral condition to tease out this confound. We would also like to note here that auditory-stimuli confounds are a common challenge when developing studies investigating effects of mindfulness. As such, we reran the model excluding the ROIs that overlapped with areas of auditory processing, and the effects remained.

Due to the small sample size of this study (N = 32), we were unable to investigate sex differences during mindfulness. There are known sex differences in emotional regulation, and brain network dynamics that support emotional regulation are dependent on fluctuating hormones across the menstrual cycle [68]. Subnetworks like the DMN and SN have varied dynamical complexity throughout the menstrual cycle, such that complexity is highest in the SN during the pre-ovulatory phase and highest in the DMN during mid-luteal phases [69]. Future work should take this into account to help delineate sex effects of mindfulness as a tool for stress reduction. Lastly, due to the aggregation of both the normal- and abstained-drinking-state scans, it is difficult draw firm conclusions about the effects of stress and abstinence on the effects of mindfulness. An appropriate next step would be to design a study to test the interaction and independent effects of drinking patterns.

## 5. Conclusions

To our knowledge, this is the first study to evaluate how mindfulness-based techniques following an acute stressor in moderate-to-heavy drinkers influence brain networks employing dynamic connectivity methodology—HSMM. The use of HSMM enabled us to track changes in brain states between our experimental conditions that would have been masked by the commonly used “static” methodology. Specifically, guided mindfulness yielded higher occupancy time and transition frequency groups to states in which nodes of the SN were more active, whereas the control group exhibited higher occupancy time and transition frequency to states in which nodes of the DMN associated with rumination were more active. We also observed lower perceived stress following the recovery period among participants in the mindfulness group as compared to the control group.

The results of the current study on mindful coping following an acute stressor are the first to demonstrate this pattern of network connectivity, albeit in the context of a single, brief mindful-based intervention. A question of both conceptual and practical relevance is whether mindfulness-based training enables participants to achieve similar brain states when confronted with life stress as well as the features of training that maximize treatment efficacy.

## Figures and Tables

**Table 1:** Sample characteristics listed as mean (SD) or frequency (percentage). * *p* < 0.05 for a two-sample *t*-test comparing the average number of drinks on drinking days between the mindfulness group vs. the control group.

Demographic Characteristic	Overall (N = 32)	Mindfulness Group (N = 15)	Control Group (N = 17)
**Age**	38.2 (10.6)	38.8 (10.6)	37.6 (10.3)
**Race**			
Caucasian	28 (87.5%)	14 (93.3%)	14 (82.3%)
African American	3 (9.4%)	1 (6.7%)	2 (11.8%)
Asian	1 (3.1%)	0	1 (5.9%)
**BMI**	25.3 (4.4)	24.2 (3.4)	26.2 (5.0)
**Sex**			
Male	13 (40.6%)	7 (46.7%)	6 (35.3%)
Female	19 (59.4%)	8 (53.3%)	11 (64.7%)
**Alcohol Use**			
Total tears drinking	18.1 (11.1)	20.0 (12.7)	16.5 (9.6)
**TLFB—Alcohol Use**			
Percentage drinking days in the past month	80.1 (16.1)	77.6 (14.1)	82.3 (17.8)
Average number of drinks on drinking days *	2.2 (0.7)	1.9 (0.5)	2.5 (0.8)
Age of first drink	19.03 (4.6)	18.5 (2.4)	20 (5.8)

Items in bold are to distinguish subheadings for demographic characteristics.

**Table 2:** Permutation test results for each state and each dynamic property. * *p* < 0.05 for a permutation test comparing mindfulness vs. control groups. All significant results survived false discovery rate (FDR) correction at a family-wise error rate of 0.05.

State	Occupancy Time	Sojourn/Dwell Time	Transition Frequency
1	0.0002 *	<0.0001 *	0.0002 *
2	0.0002 *	<0.0001 *	0.0002 *
3	0.0002 *	0.004 *	0.0002 *
4	0.2416	0.858	0.1129
5	0.5461	0.406	0.6335
6	0.8897	0.29	0.3766

**Table 3:** Results of mixed-effect model. State 2 had a significant interaction for state occupancy time, so a stratified analysis was performed on the mindfulness and control groups separately. All significant results survived false discovery rate (FDR) correction at a family-wise error rate of 0.05. * *p* < 0.05.

*Predictors (Stress Score Is the Outcome Variable)*	*b*	*Std. Error*	*t*	*p-Value*
**State 1**				
Intercept	2.6203	0.7725	3.3921	0.0007
Occupancy Time (OT)	0.0007	0.0231	0.0282	0.9775
Group Assignment (ref = Mindfulness)	0.9267	0.5737	1.6152	0.1063
Average Number of Drinks	−0.5974	0.3534	−1.6903	0.0910
Interaction (OT*Group)	0.0022	0.0233	0.0926	0.9262
**State 2**				
**Control**				
Intercept	4.5888	1.1297	4.0619	0.00005
Occupancy Time	−0.0633	0.0205	−3.0895	**0.0020** *
Average Number of Drinks	−0.7292	0.4065	−1.7934	0.0728
**Mindfulness**				
Intercept	3.7844	1.4981	2.5261	0.0115
Occupancy Time	0.0044	0.0038	1.1578	0.2469
Average Number of Drinks	−1.4778	0.8571	−1.7243	0.0847
**State 3**				
Intercept	2.4406	0.7571	3.2237	0.0013
Occupancy Time	0.0781	0.1007	0.7757	0.4379
Group Assignment (ref = Mindfulness)	0.9215	0.5530	1.6663	0.0957
Average Number of Drinks	−0.0738	0.1007	−0.7325	0.4639
Interaction (OT*Group)	−0.0738	0.1007	−0.7325	0.4639
**State 4**				
Intercept	2.004	0.7678	2.6104	0.0090
Occupancy Time	0.0054	0.0035	1.5195	0.1286
Group Assignment (ref = Mindfulness)	1.3199	0.5148	2.5638	0.0104
Average Number of Drinks	−0.4235	0.3290	−1.2871	0.1981
Interaction (OT*Group)	−0.0053	0.0055	−0.9649	0.3345
**State 5**				
Intercept	2.9269	0.8264	3.5419	0.0004
Occupancy Time	−0.0056	0.0048	−1.1678	0.2429
Group Assignment (ref = Mindfulness)	1.079	0.5910	1.8268	0.0677
Average Number of Drinks	−0.5895	0.3421	−1.7231	0.0849
Interaction (OT*Group)	0.0018	0.0058	0.3206	0.7485
**State 6**				
Intercept	3.0799	0.7918	3.8899	0.0001
Occupancy Time	−0.0115	0.0072	−1.5983	0.1099
Group Assignment (ref = Mindfulness)	0.8773	0.6197	1.4158	0.1568
Average Number of Drinks	−0.6019	0.3475	−1.7321	0.0832
Interaction (OT*Group)	0.0067	0.0089	0.7502	0.4531

Italics have been used to indicate the outputs of the mixed effects model.

## Data Availability

The data presented in this study has not been made publicly available due to a lack of informed consent to do so. However, the data can be made publicly available upon request to the authors with appropriate Institutional Review Board approval and data use agreement. The toolbox and associated code can be made available upon request.

## Supplementary Materials

The following supporting information can be downloaded at: https://www.mdpi.com/article/10.3390/brainsci16030312/s1, Figure S1: Bar plot depicting mean ± SD of perceived stress scores; Figure S2: Plot depicting the minimum distance between state pairs for each run; Table S1: ROIs used for the current study as well as the subnetwork assignment; Figure S3: Subject state sequences of both groups during their active/passive recovery scan; Figure S4: Module maps for each of the six states with the outlier included, represented by color grouping; Figure S5: Mean activation maps for each of the six states with the outlier included; Table S2: Permutation test results for each state and each dynamic characteristic with the original outlier included; Table S3: Results of mixed effect model in which occupancy time is a predictor of stress scores (with original outlier included); Table S4: Results of mixed effect model in which transition frequency is a predictor of stress scores (with original outlier included).

## Abbreviations

The following abbreviations are used in this manuscript:

NSDUH	National Survey of Drug Use and Health
NIAAA	National Institute on Alcohol Abuse and Alcoholism
DMN	Default mode network
SN	Salience network
vmPFC	Ventromedial prefrontal cortex
PCC	Posterior cingulate cortex
dACC	Dorsal anterior cingulate cortex
MBSR	Mindfulness-based stress reduction techniques
BOLD	Blood oxygenation level dependent
MRI	Magnetic resonance imaging
AUDIT	Alcohol Use and Disorders Identification Test
AUD	Alcohol use disorder
BMI	Body mass index
BAC	Blood alcohol content
TLFB	Timeline follow back
SPM	Statistical parametric mapping
HSMM	Hidden semi-Markov model
ROI	Region of interest
KL	Kullback–Leibler
dmPFC	Dorsomedial prefrontal cortex
vPFC	Ventral prefrontal cortex
CEN	Central executive network
GSR	Galvanic skin response
